# Redefining the ontogeny of hyalocytes as yolk sac-derived tissue-resident macrophages of the vitreous body

**DOI:** 10.1186/s12974-024-03110-x

**Published:** 2024-07-03

**Authors:** Dennis-Dominik Rosmus, Jana Koch, Annika Hausmann, Aude Chiot, Franz Arnhold, Takahiro Masuda, Katrin Kierdorf, Stefanie Marie Hansen, Heidrun Kuhrt, Janine Fröba, Julian Wolf, Stefaniya Boneva, Martin Gericke, Bahareh Ajami, Marco Prinz, Clemens Lange, Peter Wieghofer

**Affiliations:** 1 Institute of Anatomy, Leipzig University, 04103 Leipzig, Germany; 2 Cellular Neuroanatomy, Institute of Theoretical Medicine, Augsburg University, Universitätsstrasse 2, 86159 Augsburg, Germany; 3 Institute of Neuropathology, Medical Center, Faculty of Medicine, University of Freiburg, 79106 Freiburg, Germany; 4 Eye Center, Medical Center, Faculty of Medicine, University of Freiburg, 79106 Freiburg, Germany; 5 Department of Molecular Microbiology and Immunology, Oregon Health & Science University, Portland, OR 97239 USA; 6 Department of Behavioral Neuroscience, Oregon Health & Science University, Portland, OR 97239 USA; 7 Division of Molecular Neuroimmunology, Medical Institute of Bioregulation, Kyushu University, Fukuoka, 812-8582 Japan; 8 Centre for Integrative Biological Signalling Studies, University of Freiburg, 79106 Freiburg, Germany; 9 Centre for Basics in NeuroModulation (NeuroModulBasics), Faculty of Medicine, University of Freiburg, 79106 Freiburg, Germany; 10 Molecular Surgery Laboratory, Stanford University, Palo Alto, CA 94304 USA; 11 Department of Ophthalmology, Byers Eye Institute, Stanford University, Palo Alto, CA 94304 USA; 12 Signalling Research Centres BIOSS and CIBSS, University of Freiburg, 79106 Freiburg, Germany; 13 Ophtha Lab, Department of Ophthalmology, St. Franziskus Hospital, 48145 Münster, Germany

**Keywords:** Macrophages, Hyalocytes, Vitreous body, Cx3cr1, Fate mapping, Turnover, Development, CSF1R

## Abstract

**Background:**

The eye is a highly specialized sensory organ which encompasses the retina as a part of the central nervous system, but also non-neural compartments such as the transparent vitreous body ensuring stability of the eye globe and a clear optical axis. Hyalocytes are the tissue-resident macrophages of the vitreous body and are considered to play pivotal roles in health and diseases of the vitreoretinal interface, such as proliferative vitreoretinopathy or diabetic retinopathy. However, in contrast to other ocular macrophages, their embryonic origin as well as the extent to which these myeloid cells might be replenished by circulating monocytes remains elusive.

**Results:**

In this study, we combine transgenic reporter mice, embryonic and adult fate mapping approaches as well as parabiosis experiments with multicolor immunofluorescence labeling and confocal laser-scanning microscopy to comprehensively characterize the murine hyalocyte population throughout development and in adulthood. We found that murine hyalocytes express numerous well-known myeloid cell markers, but concomitantly display a distinct immunophenotype that sets them apart from retinal microglia. Embryonic pulse labeling revealed a yolk sac-derived origin of murine hyalocytes, whose precursors seed the developing eye prenatally. Finally, postnatal labeling and parabiosis established the longevity of hyalocytes which rely on Colony Stimulating Factor 1 Receptor (CSF1R) signaling for their maintenance, independent of blood-derived monocytes.

**Conclusion:**

Our study identifies hyalocytes as long-living progeny of the yolk sac hematopoiesis and highlights their role as integral members of the innate immune system of the eye. As a consequence of their longevity, immunosenescence processes may culminate in hyalocyte dysfunction, thereby contributing to the development of vitreoretinal diseases. Therefore, myeloid cell-targeted therapies that convey their effects through the modification of hyalocyte properties may represent an interesting approach to alleviate the burden imposed by diseases of the vitreoretinal interface.

**Supplementary Information:**

The online version contains supplementary material available at 10.1186/s12974-024-03110-x.

## Background

Macrophages are highly versatile immune cells residing in different tissues and compartments of the body with important roles in the host’s innate immunity. Originally thought to be descendants of the “mononuclear phagocyte system” [1], the last decade of research uncovered a remarkable heterogeneity of tissue-resident macrophages in terms of their origin, fate and function in homeostasis and disease [2, 3]. Today, it is commonly accepted that myeloid cells arise from different hematopoietic waves during pre- and postnatal development [4, 5], constituting a highly specialised network of myeloid cells adapted to their respective local niches due to environmental cues [6]. Studies in rodents revealed that the first tissue-resident macrophages originate from early erythromyeloid precursors (EMPs) in the extra-embryonic yolk sac (YS) at embryonic day 8.5 (E8.5) [7–10]. EMPs further differentiate into an A1 and a CX_3_CR1-expressing A2 population which in turn seed prenatal tissue-resident macrophage populations in nearly all tissues [11–13] including microglia, extraparenchymal CNS-associated macrophages (CAMs) [11, 14–16] and ocular macrophages [17–19]. It is assumed that a later wave of EMPs is generated in the YS. These EMPs subsequently colonize the fetal liver (FL) between E8.5-E10.5 giving rise to EMP-derived FL monocytes that outcompete YS-derived macrophages in the majority of organs [2, 20]. Finally, hematopoietic stem cells (HSCs) develop in the aorto-gonad-mesonephros region around E10.5 and establish the intra-embryonic definitive hematopoiesis which persists until adulthood [21, 22].

The murine eye is composed of different compartments with resident myeloid cell populations. Recent studies using different fate mapping approaches demonstrated that nearly all ocular compartments harbor prenatally seeded macrophage populations [17, 18]. While retinal microglia (rMG) derive from the YS and are maintained by self-renewal, macrophages in the ciliary body, cornea and the choroid have a dual origin with varying degrees of replenishment by peripheral monocytes during adulthood [17, 18]. Hyalocytes, however, have long been overlooked with respect to their origin, heterogeneity, and function as tissue-resident macrophages of the vitreous [23]. Since their first description in the nineteenth century [24], hyalocytes have been implicated in a variety of physiological and pathological processes [23, 25–27]. In the healthy eye, hyalocytes participate in the synthesis of extracellular matrix components and are believed to contribute to the maintenance of the ocular immune privilege [25, 28]. Furthermore, hyalocytes are highly dynamic cells and are regularly distributed above the inner limiting membrane (ILM) as shown by optical coherence tomography in humans [29, 30]. The ILM represents a barrier consisting of distinct laminae that are built by both the vitreous providing a dense meshwork of collagen fibrils and the retina abutting the vitreal side via the retinal glia limitans built by endfeet of Mueller glia cells and astrocytes inhabiting the nerve fiber and ganglion cell layer [31]. The glia limitans is connected to the abluminal side of the vitreal laminae via extracellular matrix components. Hence, rMG reside in the CNS while hyalocytes are located close to the border of the CNS and thus colonize distinct compartments of the vitreoretinal interface [32, 33].

Previous studies addressed the origin of hyalocytes using bone marrow chimeras as a tool to investigate the contribution of adult hematopoiesis to resident myeloid cell populations and found a nearly complete replenishment of the resident hyalocyte population by GFP^+^ donor cells [23, 34]. However, the experimental paradigm of bone marrow chimerism often requires irradiation-induced depletion of the host’s bone marrow as a precondition, which can cause changes in vascular structures and immunological barrier breakdowns. These may facilitate the engraftment of monocyte-derived macrophages in the CNS [35–37] including the retina [38], which does not occur under physiological conditions. Therefore, the main objective of our study was to scrutinize the previously established origin of murine hyalocytes and to determine their postnatal turnover. To achieve this goal, we utilized transgenic reporter mouse lines, modern fate mapping tools as well as parabiosis techniques. We demonstrate that, contrary to current conceptions, hyalocytes are mainly derived from precursors of the extra-embryonic YS, represent a long-lived self-maintaining cell population independent of adult hematopoiesis, and rely on functional Colony Stimulating Factor 1 Receptor (CSF1R) signaling as a prerequisite for their maintenance. In summary, these data characterize murine hyalocytes as YS-derived, tissue-resident macrophages that may substantially contribute to physiological and pathological processes at the vitreoretinal interface.

## Materials and methods

### Mice

In this study, C57BL/6 J mice (RRID: IMSR_JAX:000664) were used as wildtype (WT) mice. All transgenic lines including *Actb*^*GFP/*+^ (RRID: IMSR_JAX:006567), *Ubc*^*GFP/*+^ (RRID: IMSR_JAX:004353)*, Cx3cr1*^GFP/+^ (RRID: IMSR_JAX:005582), *Tmem119*^*GFP/*+^ (RRID: IMSR_JAX:031823), *Hexb*^*tdT/tdT*^ (kindly provided by Dr. Takahiro Masuda and Prof. Dr. Marco Prinz), *Cx3cr1*^*CreER*^ (RRID: IMSR_JAX:020940), *Csf1r*^*EGFP/*+^ (RRID: IMSR_JAX:018549), *Csf1r*^*∆FIRE/∆FIRE*^ (RRID: IMSR_JAX:032783)*, Flt3*^*Cre*^ (RRID: IMSR_EM:11790), and *Rosa26*^fl−stop−fl−EYFP^ (*Rosa26*-YFP, RRID: IMSR_JAX:006148) were bred on a C57BL/6 J background. All mice were housed in a 12-h light–dark cycle with ad libitum access to food and water and were kept under specific pathogen-free conditions and devoid of *Crb1* (RD8) mutations. *Cx3cr1*^*CreER*^ mice were crossed to *Rosa26*-YFP mice to generate *Cx3cr1*^*CreER*/+^:*Rosa26-YFP*^fl/fl^ animals that were identified by PCR screening. *Flt3*^*Cre*^ mice were crossed to *Rosa26-YFP* mice.

### Tamoxifen (TAM) treatment

To induce nuclear CreER recombinase activity and expression of YFP in adult *Cx3cr1*^*CreER*^*:Rosa26-YFP* mice, six-week-old mice were injected subcutaneously with 4 mg of TAM (T5648-1G, Sigma-Aldrich, Taufkirchen, Germany) dissolved in corn oil (C8267, Sigma-Aldrich, Taufkirchen, Germany) at 20 mg/ml. Overall, three injections were performed, one every two days. To confirm recombination, blood was collected from the retro-orbital sinus one week after the last injection, and YFP expression of immune cells was assessed by flow cytometry. At 2 or 26 weeks post-injections, mice were deeply anesthetized with ketamine (West-Ward Pharmaceuticals) and transcardially perfused with 0.1 M ice-cold phosphate buffered saline (PBS). Blood and one cerebral hemisphere per mouse were collected to assess YFP percentage of different cell populations by flow cytometry. Both eyes were collected and post-fixed in 4% paraformaldehyde (PFA) for 1 h at room temperature (RT) for histological procedures. For embryonic pulse labeling experiments, pregnant females at day 9 post-coitum underwent intraperitoneal (i.p.) injections with 200 µl of 20 mg/ml TAM (T5648-1G, Sigma-Aldrich, Taufenkirchen, Germany) and 10 mg/ml Progesterone (P0130, Merck) dissolved in corn oil (C8267, Sigma-Aldrich, Taufenkirchen, Germany).

### BM transplantation

Eight-week-old recipient wildtype mice (*Actb*^+/+^) were irradiated and reconstituted by injecting 5 × 10^6^ bone marrow cells derived from the femur and tibia of adult donor (*Actb*^*GFP/*+^) mice into the tail vein of recipients. Mice received whole-body irradiation (11 Gy) 24 h prior to bone marrow reconstitution with an RS 2000 Biologica x-Ray Irradiator. After 4 weeks, reconstitution efficiency was assessed by flow cytometry and was found to be > 90% in Ly6C^hi^ monocytes.

### Parabiosis

Six- to seven-week-old pairs of weight-matched C57BL/6 J WT and *Ubc*^GFP/+^ mice were surgically joined as previously described [35]. Prior to surgery, mice were housed together for two weeks and provided with soft food. To confirm blood sharing, blood was collected from the tail vein, and GFP expression was assessed by flow cytometry two weeks post-surgery. At 4 or 28 weeks post-surgery (i.e. 2 weeks and 26 weeks after establishment of blood sharing), mice were deeply anesthetized with ketamine (West-Ward Pharmaceuticals) and transcardially perfused with 0.1 M ice-cold PBS. Blood, spleen and one cerebral hemisphere per mouse were collected to assess GFP percentage of cells by flow cytometry. Both eyes were collected and post-fixed in 4% PFA for 1 h at RT for histological procedures.

### Immunofluorescence

After transcardial perfusion with PBS followed by 4% PFA, eyes were fixed in 4% PFA for 1 h at RT and processed either for flat mounts as previously described [39] or cryosectioning. Briefly, eye cups were incubated consecutively in 10%, 20%, 30% sucrose solution after fixation and embedded in Tissue-Tek® O.C.T.TM Compound (Sakura Finetek Germany GmbH). 12 µm sections or retinal flat mounts were blocked with PBS containing 2% (cryosections) or 1% (retinal flat mounts) bovine serum albumin (BSA), 0.1% Triton-X 100 and 2% normal goat or donkey serum, respectively. Primary antibodies were added overnight at a dilution of 1:500 for Iba-1 (234 013, Synaptic Systems, Göttingen, Germany, RRID: AB_2661873; 234 004, Synaptic Systems, Göttingen, Germany, RRID: AB_2493179), 1:1000 for anti-GFP (R1091P, Acris Antibodies Inc., San Diego, USA, RRID: AB_1002036), 1:100 for F4/80 (MF48000, Invitrogen, Waltham, USA, RRID: AB_10376289), 1:500 for TMEM119 (400 002, Synaptic Systems, Göttingen, Germany, RRID: AB_2721104), 1:100 for MHC class II (107 602, BioLegend, USA, RRID: AB_313317), 1:100 for CD206 (ab64693, abcam, UK, RRID: AB_1523910), 1:100 for CD163 (155 302, BioLegend, USA, RRID: AB_2734239), 1:250 for LYVE1 (AF2125, R&D Systems, USA, RRID: AB_2297188), 1:800 for CD11b (ab64347, abcam, UK, RRID: AB_1140550), and 1:200 for Collagen IV (AB769, Millipore, USA, RRID: AB_92262). For the visualization of myeloid cells and blood vessels, rhodamine-labeled Isolectin-B4 from *Griffonia simplicifolia* (RL-1102, Vector Laboratories, RRID: AB_2336492) was used. After washing three (cryosections) or five (retinal flat mounts) times with washing buffer containing 2% (cryosections) or 1% (retinal flat mounts) BSA, 0.1% Triton X-100 and 0.2% normal goat or donkey serum, secondary antibodies were added at a dilution of 1:500 (Alexa Fluor® 488, Alexa Fluor® 568 and Alexa Fluor® 647, Thermo Fisher Scientific, Waltham, USA) for 2 h at RT (cryosections) or overnight at 4 °C (retinal flat mounts). The following secondary antibodies were used in this study: Goat anti-rabbit Alexa Fluor® 488 (A11034, Invitrogen, USA, RRID: AB_2576217), Goat anti-rat Alexa Fluor® 568 (A11077, Invitrogen, USA, RRID: AB_2534121), Goat anti-rabbit Alexa Fluor® 647 (A21245, Invitrogen, USA, RRID: AB_2535813), Goat anti-guinea pig Alexa Fluor® 647 (A21450, Invitrogen, USA, RRID: AB_141882), Donkey anti-rabbit Alexa Fluor® 568 (A10042, Invitrogen, USA, RRID: AB_2534017), and Donkey anti-goat Alexa Fluor® 647 (A21447, Invitrogen, USA, RRID: AB_2535864). Nuclei were counterstained with 4′,6-Diamidine-2-phenylindole (DAPI) at a dilution of 1:10,000 for 10 min followed by extensive washing with PBS. Images were taken using a conventional fluorescence microscope (Olympus BX-61 with a color camera (Olympus DP71)) (Olympus, Tokyo, Japan) or a confocal microscope (Olympus Fluoview FV 1000 using a 20 × 0.95 NA XLUMPlanFL N, 20 × 0.75 NA U Plan S Apo or a 40 × 0.95 NA U Plan S Apo objective or Leica Stellaris 5 using a HC PL APO 20x/0.75 CS2). For confocal z-stacks, a z-step size of 1.5 µm was chosen and images were taken from the retinal surface above the inner limiting membrane to the inner plexiform layer. Confocal z-stacks were subsequently used to generate projection images using maximum intensity projections over the z-axis.

### Flow cytometry

For isolation of microglial cells, brains were dissected and cut in half along the longitudinal fissure. One cerebral hemisphere per animal was homogenized with a dounce homogenizer in 0.1 M HBSS (Gibco), filtered through a 70 µm cell strainer (Falcon) and centrifuged (750 g, 7 min, 4 °C) (Thermo Scientific). To remove the myelin, cells were resuspended in 5 ml of 0.1 M HBSS + 2 ml of RPMI (Gibco) + 3 ml of isotonic Percoll (ISP) (90% Percoll (GE Healthcare) + 10% 1 M HBSS). A 2 ml layer of 70% Percoll (70% ISP, 30% 0.1 M HBSS) was added at the bottom of the tube using spinal cord needles (BD Biosciences). Cells were centrifuged at 500 g for 15 min at RT without break. Following centrifugation, myelin was removed from the top layer, and cells were recovered from the interface between the 30% and 70% Percoll layers. Cells were washed twice using 0.1 M PBS (Gibco) supplemented with 2% fetal bovine serum (FBS) (Atlas Biologicals) followed by antibody staining. Blood was collected in a heparin tube (BD Vacutainer) from cardiac puncture when the mouse was sacrificed. Approximately 50 µl of blood was transferred to a 15-ml conical tube. Erythrocytes from blood samples were lysed in 2 ml of 10X RBC lysis buffer diluted 1:10 in water (eBioscience) for 7 min at RT. Blood samples were then washed two times in 0.1 M PBS supplemented with 2% FBS, followed by staining.

### Validation of recombination efficiency in *Cx3cr1*^*CreER*^*:Rosa26-YFP* mice

White blood cell and microglial cell Fc-receptors were blocked using a purified anti-CD16/CD32 antibody (1:200, clone 93, BioLegend, USA, RRID: AB_312807) for 20 min at 4 °C. White blood cells were stained for 30 min at 4 °C using a viability dye (1:2000, Ghost Dye Red 780, TONBO) in addition to the following antibodies: CD11b-BV421 (1:200, clone M1/70, BioLegend, USA, RRID: AB_10897942), CD115-BV605 (1:200, clone AFS98, BioLegend, USA, RRID: AB_2562760), CD45-BUV395 (1:200, clone 30-F11, BD Biosciences, USA, RRID: AB_2651134) and Ly6C-PE (1:16,000, clone HK1.4, BioLegend, USA, RRID: AB_1732082). Brain microglial cells were stained for 30 min at 4 °C using a viability dye (1:2000, Ghost Dye Red 780, TONBO) in addition to the following antibodies: CD11b-BV421 (1:200, clone M1/70, BioLegend, USA, RRID: AB_10897942), CD45-BUV395 (1:200, clone 30-F11, BD Biosciences, USA, RRID: AB_2651134).

### Validation of a successful post-surgery shared circulation in parabiotic mice

White blood cell, splenocyte and microglial cell Fc-receptors were blocked using a purified anti-CD16/CD32 antibody (1:200, clone 93, BioLegend, USA, RRID: AB_312807) for 20 min at 4 °C. White blood cells and splenocytes were then stained for 30 min at 4 °C, using a viability dye (1:2000, Ghost Dye Red 780, TONBO) in addition to the following antibodies: CD11b-BV421 (1:200, clone M1/70, BioLegend, USA, RRID: AB_10897942), CD45-BUV395 (1:200, clone 30-F11, BD Biosciences, USA, RRID: AB_2651134), CD19-BUV737 (1:1600, clone 1D3, BD Biosciences, USA, RRID: AB_2870111), CD3ε-APC (1:400, clone 145-2C11, BioLegend, USA, RRID: AB_312677) and Ly6G-PE (1:200, clone 1A8, BioLegend, USA, RRID: AB_1186099). Microglial cells were stained as described above. Cells were acquired on a BD FACSymphony Cell Analyzer (BD Biosciences, USA), and a multiparameter analysis was performed using FlowJo Software Version 10.8.1 (BD Biosciences, USA).

### Statistical analysis

Using previous data as guidance [15, 18], we ensured a comparable group size for the quantitative analysis of the turnover in *Cx3cr1*^*CreER*^*:Rosa26-YFP* mice and parabiotic mice. Statistical analysis was performed using GraphPad Prism 7 (GraphPad Software, La Jolla, USA). Data were tested for normality using the Kolmogorov–Smirnov test. If normality was given, an unpaired t-test or one-way ANOVA was applied, if not indicated otherwise. If normality was not given, the Mann–Whitney or Kruskal–Wallis test was applied. Differences were considered statistically significant for a p-value < 0.05.

## Results

### Immunophenotyping of murine hyalocytes under homeostatic conditions

**Fig. 1:**
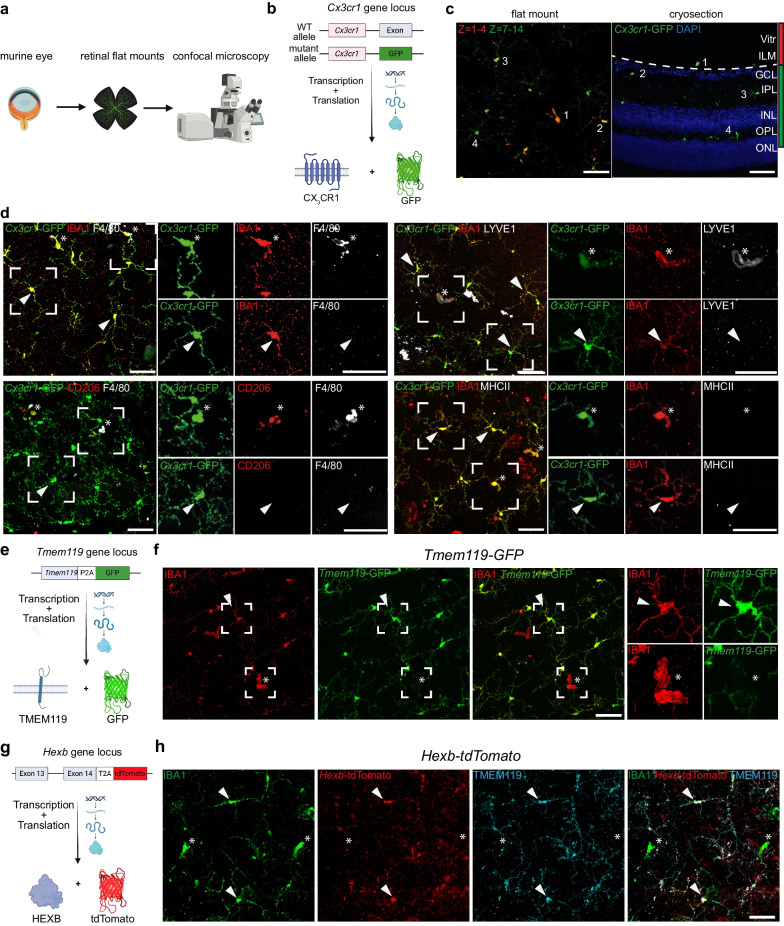
Immunophenotyping of murine hyalocytes underlines their myeloid cell Identity.** a** Graphical illustration of the experimental setup. Retinal flat mounts were prepared of eyes of several transgenic reporter mouse models and analyzed using immunofluorescence labeling and confocal laser-scanning microscopy. **b** Graphical scheme of gene targeting in *Cx3cr1-GFP* mice where one allele of the *Cx3cr1* locus is replaced by green fluorescent protein (GFP). **c** Representative images from retinal flat mount (left) and cryosection (right) from *Cx3cr1-GFP* mice depicting the anatomical localization of hyalocytes and retinal microglia (rMG). Planes 1-4 (red) include hyalocytes (1) residing above the inner limiting membrane (ILM, dashed line). Planes 7-14 (green) comprise rMG positioned in the inner plexiform layer (IPL) (2, 3) and outer plexifom layer (OPL) (4). The z- step size is 1,5 µm. Vitr: vitreous, GCL: ganglion cell layer, INL: inner nuclear layer, ONL: outer nuclear layer. **d** Representative images of retinal flat mounts from *Cx3cr1-GFP* mice. In contrast to rMG (arrowheads), murine hyalocytes (asterisks) express F4/80, CD206 and LYVE1. Both cell types did not show MHCII-immunoreactivity. Pictures are representative for three (CD206, LYVE1, MHCII) or five (F4/80) mice, respectively. **e** Graphical scheme depicting gene targeting in *Tmem119-GFP* mice. GFP was introduced in the stop codon of the *Tmem119* locus separated by a P2A-site enabling functional expression of both proteins. **f** Representative images of retinal flat mounts from *Tmem119-GFP* mice. Hyalocytes were GFP^−^ (asterisks) while rMG (arrowheads) are consistently GFP^+^. Images are representative for three mice. **g** Graphical illustration of gene targeting in *Hexb-tdTomato* reporter mice. A tdTomato cassette was introduced downstream of Exon 14 of the *Hexb* locus separated through a T2A-site enabling the expression of functional HEXB protein and tdTomato. **h** Representative images of retinal flat mounts from *Hexb-tdTomato* mice. Hyalocytes were identified as tdTomato^−^TMEM119^−^ cells (asterisks) compared to tdTomato^+^TMEM119^+^ rMG (arrowheads). Images are representative for two mice. All scale bars equal 50 µm

Previous studies have shown that hyalocytes are tissue-resident macrophages that belong to the innate immune system of the eye [40] and are widely regarded as the main cell population of the vitreous. Importantly, they are distinct from other cell types at the vitreoretinal interface such as fibroblasts [41]. However, a characterization of the marker profile of murine hyalocytes that combines modern genetic tools for the labeling of tissue-resident macrophages with multi-color immunofluorescence to unveil the immunophenotype of hyalocytes, also in relation to rMG would provide valuable insights into the relationship between those two macrophage populations residing at the vitreoretinal interface. Therefore, we took advantage of several transgenic reporter mouse lines and dissected eyes for retinal flat mounts, subsequent immunofluorescence labeling and imaging using confocal laser-scanning microscopy (Fig. 1a). First of all, we used the *Cx3cr1*^GFP/+^ reporter mouse line, in which the green fluorescent protein (GFP) is expressed under the control of the fractalkine receptor (CX_3_CR1) promoter and labels myeloid cells [42] (Fig. 1b). For the purpose of this study, we defined GFP^+^ cells located above the ILM, which represents the border between the vitreous body and the underlying neuroretina, as preretinal hyalocytes (Fig. 1c). To visualize the anatomical localization of GFP^+^ cells in relation to the ILM, we used a color-coded maximum intensity projection of distinct layers of the confocal z-stack to illustrate the depth of the tissue (Fig. 1c). We found that hyalocytes are regularly distributed above the ILM and express common myeloid cell markers such as the pan-macrophage marker IBA1 similar to microglia in the retina, but also selectively express markers distinct from microglia such as F4/80, the mannose receptor CD206 and the scavenger receptor CD163 (Fig. 1d, Additional file 1: Fig. S1). Hyalocytes were positive for LYVE1 which is typically expressed in certain subpopulations of macrophages across organs [43] and was previously identified as a marker for vitreal macrophages [44]. Interestingly, neither rMG nor hyalocytes, show any immunoreactivity for major histocompatibility complex II (MHCII) (Fig. 1d).

To further exclude the possibility that these cells may represent translocated microglia from the inner plexiform-(IPL) or ganglion cell layer (GCL), we utilized TMEM119, which belongs to the core signature genes of microglia in the brain and the retina [18, 45–48], as a marker to discriminate between microglia and hyalocytes. We analyzed retinal flat mounts from *Tmem119-GFP* mice expressing GFP under the control of the *Tmem119* promoter, without affecting the production of the native protein through polycistronic gene expression (Fig. 1e), to specifically label microglia in the CNS including the retina [49]. Indeed, hyalocytes, which were positive for IBA1 and located above the ILM in the posterior vitreous cortex, did not show any sign of GFP-labeling in comparison to rMG, which were consistently GFP-positive (Fig. 1f). Finally, we used the recently generated *Hexb-tdTomato* reporter mouse model, which enables stable gene targeting of microglia in the CNS under homeostatic and pathological conditions, to further characterize murine hyalocytes **(**Fig. 1g**)** [50]. Notably, hyalocytes did not express the *Hexb-tdTomato* transgene in contrast to rMG exhibiting a consistent tdTomato-labeling and expressing TMEM119, which further supports our findings from *Tmem119-GFP* mice (Fig. 1h). In summary, hyalocytes in the vitreous body express macrophage cell markers, but concomitantly possess a distinct immunophenotype that clearly separates them from microglia in the retina.

### Immunofluorescence labeling reveals spatial relationships of ocular macrophages during development

**Fig. 2:**
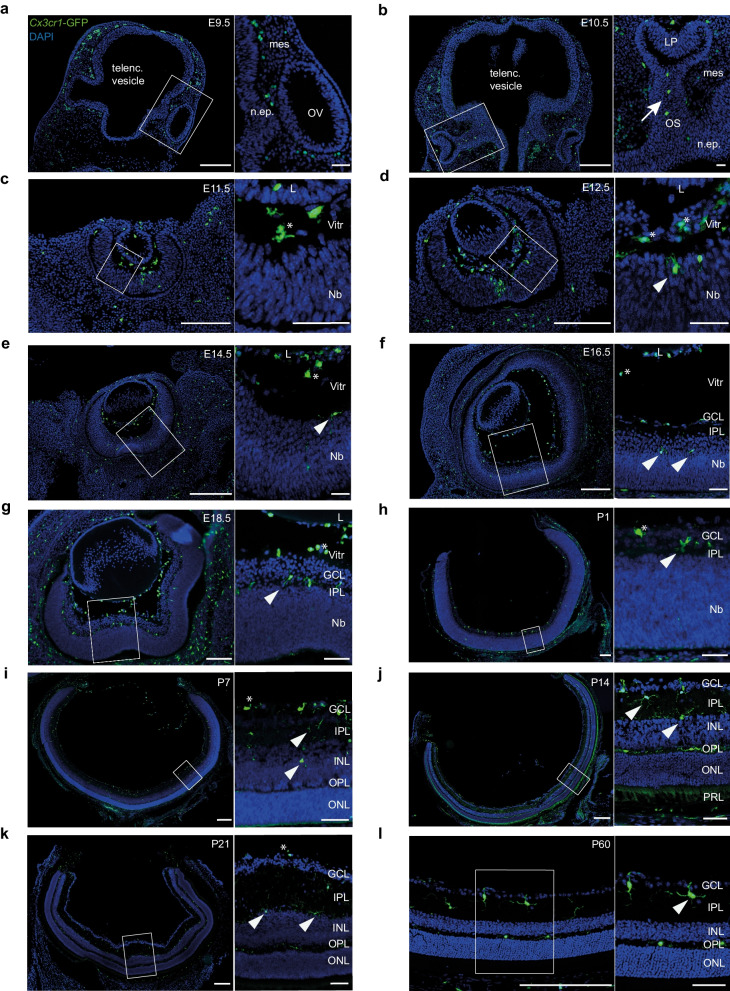
Histological analysis of the spatiotemporal development of myeloid cell populations in the murine eye. **a**–**g** Cryosections from *Cx3cr1-GFP* mice at different time points during prenatal development. GFP^+^ myeloid cells can be found in the periocular mesenchyme as early as embryonic day (E) E9.5 and enter the optic cup through the optic stalk at E10.5 (arrow), whereas hyalocytes (asterisks) can be distinguished from microglia (arrowheads) for the first time at E11.5 by localization. Scale bar = 200 µm (overview) or 50 µm (magnification). **h–l** Representative images from cryosections of *Cx3cr1-GFP* mice during early postnatal development. Hyalocytes can be found in the vitreous (asterisk), retinal microglia (arrowheads) are evenly distributed across the emerging plexiform layers. Scale bar = 200 µm (overview) or 50 µm (magnification). *telenc. vesicle* telencephalic vesicle, *mes*—mesenchyme, *n.ep.*—neuroepithelium, *OV*—optic vesicle, *OS*—optic stalk, *LP*—lens placode, *L*—lens,* Nb*—neuroblast layer, *Vitr*—vitreous, *GCL*—ganglion cell layer, *IPL*—inner plexiform layer, *INL*—inner nuclear layer, *OPL*—outer plexiform layer, *ONL*—outer nuclear layer, *PRL*—photoreceptor layer. Images are representative for n = 2 mice per time point

The embryonic development of the eye is a highly sophisticated process orchestrated by different regulatory mechanisms which collectively lead to the establishment of this complex organ [51]. To get deeper insights into the formation of the resident hyalocyte population, our next step was to perform a comprehensive histological analysis of these cells at different stages of pre- and postnatal development. Using *Cx3cr1-GFP* reporter mice, we found that myeloid cells emerge as early as E9.5 in the mesenchyme (mes) surrounding the developing optic vesicle (OV) consisting of neuroepithelium (n.ep.), which represents an evagination of the diencephalon in the forebrain [52, 53] (Fig. 2a). At E10.5, we observed GFP^+^ myeloid cells entering the optic cup, which develops from the OV by invagination during the formation of the lens placode (LP) [51]. The optic cup is part of the diencephalon, connected to this part of the developing CNS through the optic stalk (OS) in which GFP^+^ cells can be found (Fig. 2b) [51]. At E11.5, E12.5, and E14.5, GFP^+^ myeloid cells can be found in the developing neuroretina in close proximity to the neuroblast (Nb) layer, most likely destined to mature to rMG. On the other side, GFP^+^ cells found in the vitreous body (Vitr) near the neuroretina and the lens (L) vesicle likely represent early hyalocytes (Fig. 2c, 2d, 2e), which express CD11b and F4/80 at the protein level, and can be identified via Isolectin-B4 labeling, which was previously found to bind to microglial cells in the CNS [54] (Additional file 2: Fig. S2). At E16.5 and E18.5, the differentiation of the GCL and the IPL, where most of the rMG can be found [55], characterizes neuroretinal development. At this stage, GFP^+^ hyalocytes are found in the vitreous and close to the posterior pole of the lens (L) (Fig. 2f, 2g). During postnatal development, microglia are regularly distributed across the IPL and outer plexiform layer (OPL) (Fig. 2h, 2i, 2j, 2k, 2l), whereas hyalocytes reside in the vitreous body (Fig. 2h, 2i, 2k). Taken together, these stainings reveal that hyalocytes localize in the developing vitreous of the eye as early as at E11.5 and are in close vicinity to the neuroretina and the lens during prenatal development.

### Embryonic pulse labeling establishes a prenatal origin of murine hyalocytes

**Fig. 3:**
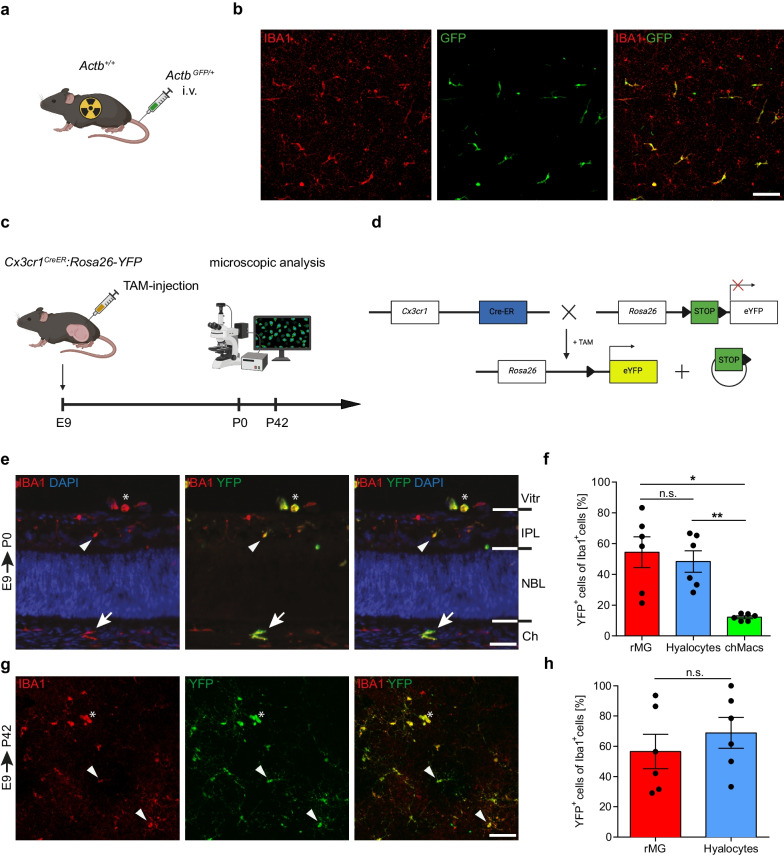
Embryonic pulse labeling reveals a prenatal origin of murine hyalocytes. **a** Schematic of bone marrow chimera creation. Wildtype mice (*Actb*^+*/*+^) were whole-body irradiated and reconstituted intravenously (i.v.). with bone marrow of *Actb*^*GFP/*+^ mice. **b** Images of retinal flat mounts of *Actb*^*GFP/*+^*:Actb*^+*/*+^ bone marrow chimeras. GFP^+^ donor-derived hyalocytes are present in the vitreous. Images are representative for three mice. Scale bar = 100 µm. **c** Schematic of embryonic pulse labeling experiment. *Cx3cr1*^*CreER*^*:Rosa26-YFP* mice were injected with tamoxifen (TAM) at embryonic day (E) 9 and analyzed at postnatal day (P) 0 or P42. **d** Illustration of embryonic pulse labeling approach. TAM administration activates inducible Cre-recombinase which irreversibly removes a LoxP-site-flanked STOP-sequence in *Cx3cr1*-expressing cells, causing consistent YFP-expression and labeling CX_3_CR1^+^ cells and their progeny. **e** Images of E9.0-labeled hyalocytes (asterisk), rMG (arrowhead) and choroidal macrophages (chMacs) (arrow) in a cryosection from *Cx3cr1*^*CreER*^*:Rosa26-YFP* mice at P0. Images are representative for six mice from three independent experiments. Scale bar = 50 µm. *Vitr*—vitreous, *IPL*—inner plexiform layer, *NBL*—neuroblast layer, *Ch*—choroid. **f** Quantification of YFP^+^ cells among IBA1^+^ hyalocytes, rMG and chMacs in E9.0-labeled *Cx3cr1*^*CreER*^*:Rosa26-YFP* mice at P0. Graphs depict mean $\pm$ S.E.M for six mice from three independent experiments. Statistics: one-way repeated measure ANOVA with post-hoc Tukey’s multiple-comparison test (* *p* < *0.05,* ** *p* < *0.01*). **g** Images of retinal flat mounts depicting E9.0-labeled hyalocytes (asterisk) and rMG (arrowheads) from *Cx3cr1*^*CreER*^*:Rosa26-YFP* mice at P42. Images are representative for six mice. Scale bar = 50 µm. **h** Percentage of YFP^+^ cells among IBA1^+^ hyalocytes and rMG in E9.0-labeled *Cx3cr1*^*CreER*^*:Rosa26-YFP* mice at P42. Graphs depict mean $\pm$ S.E.M for six mice. Statistics: paired t-test (n.s., *p* > *0.05)*

The investigation of the origin and fate of murine hyalocytes in the past has largely been restricted to lethally irradiated and bone marrow-reconstituted mice that are consequently affected by an artificial engraftment of peripheral monocytes into the tissue [18, 23, 35–38]. Indeed, we found that reconstitution of whole-body irradiated wildtype mice by intravenous injection of bone marrow obtained from *Actb*^*GFP/*+^ animals (Fig. 3a) led to an engraftment of GFP^+^ cells in the hyalocyte pool (Fig. 3b). These IBA1^+^GFP^+^ cells were located in the posterior vitreous cortex above the ILM and resembled hyalocytes under homeostatic conditions (Fig. 3b). To circumvent the use of artifact-causing irradiation, we utilized the *Cx3cr1*^*CreER*^*:Rosa26-YFP* mouse line in an embryonic pulse labeling approach as described previously (Fig. 3c) [15, 18, 56, 57]. In this model, injection of tamoxifen (TAM) at E9.0 leads to nuclear translocation of the Cre-ER fusion protein in CX_3_CR1^+^ A2 progenitors in the extra-embryonic YS, that give rise to tissue-resident macrophages in various prenatal organs including the brain, eye, liver, and lung [11–13, 18, 58, 59], and subsequent labeling of these cells and their progeny (Fig. 3d). Using this approach, we identified YFP^+^ hyalocytes in mice at postnatal day 0 (P0) (Fig. 3e), confirming the contribution of YS-derived progenitors to the hyalocyte population. Subsequent quantitative analysis of hyalocytes, rMG and macrophages in the choroid at P0 revealed that hyalocytes exhibited similar CreER-induced labeling or recombination rates (48.35 ± 7.01%) to rMG (54.42 ± 10.06%) (Fig. 3f), while the recombination rates in choroidal macrophages were significantly lower (12.13 ± 0.91%) (Fig. 3f). In order to extend our analysis over the perinatal period, we analyzed *Cx3cr1*^*CreER*^*:Rosa26-YFP* mice that were exposed to TAM treatment at E9.0 and sacrificed at P42. In support of our data at P0, we found a consistent YFP-labeling of rMG and hyalocytes (Fig. 3g) with hyalocytes (68.91 ± 10.30%) displaying similar recombination rates in comparison to rMG (56.56 ± 11.33%) (Fig. 3h).

In summary, our embryonic pulse labeling approach establishes a prenatal origin of murine hyalocytes and points towards the extra-embryonic YS as the major source.

### Postnatal fate mapping identifies hyalocytes as long-living tissue-resident macrophages

**Fig. 4:**
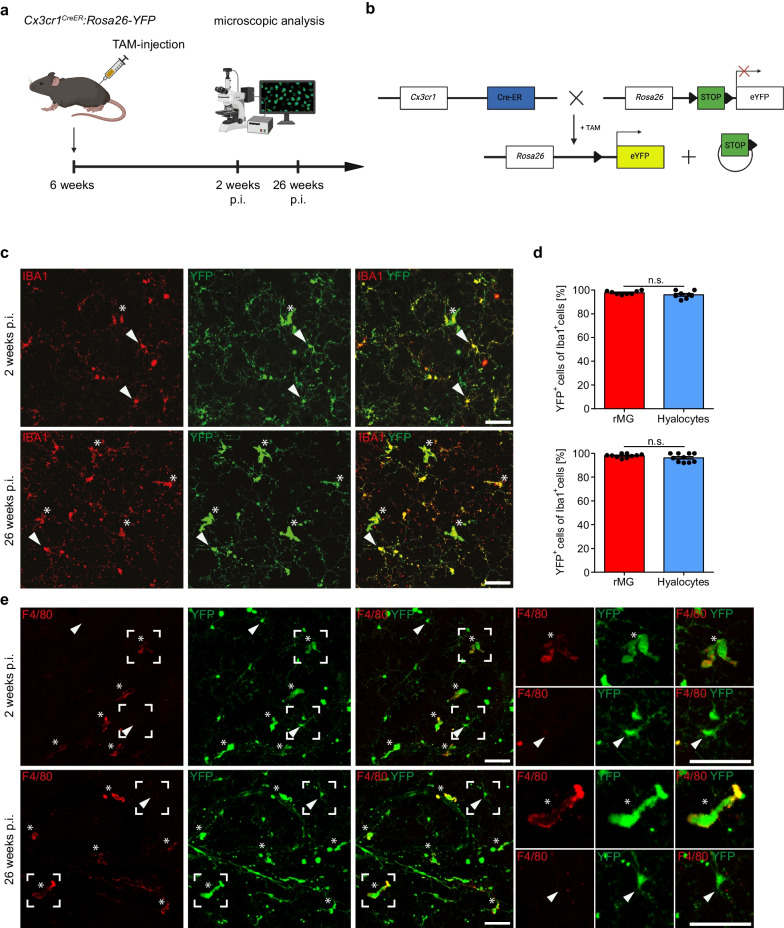
Hyalocytes represent a long-living tissue-resident macrophage population.** a** Graphical scheme of the experimental setup. Six-week-old *Cx3cr1*^*CreER*^*:Rosa26-YFP* mice were injected with tamoxifen (TAM) and retinal whole mounts subsequently analyzed by fluorescence microscopy at 2 and 26 weeks post-injection (p.i.). **b** Graphical scheme illustrating the rationale of the adult turnover approach. TAM administration leads to nuclear translocation of cytosolic Cre-ER fusion protein and subsequent Cre-mediated irreversible excision of a LoxP-site-flanked STOP-cassette in *Cx3cr1*-expressing cells. This causes a consistent level of YFP-expression under the control of the constitutively active *Rosa26* promoter and labeling of CX_3_CR1-positive cells and their progeny. **c** Confocal images of YFP^+^ and IBA1^+^ hyalocytes (asterisks) and microglia (arrowheads) in *Cx3cr1*^*CreER*^*:Rosa26-YFP* at 2 weeks and 26 weeks after injection of TAM. Images are representative for eight animals (2 weeks) from three independent experiments and ten animals (26 weeks) from two independent experiments, respectively. Scale bar = 50 µm. **d** Percentages of YFP^+^ cells among IBA1^+^ hyalocytes and rMG in *Cx3cr1*^*CreER*^*:Rosa26-YFP* mice 2 weeks (upper plot, N = 8, n.s., p > 0.05, paired t-test) and 26 weeks (lower plot, N = 10, n.s., p > 0.05, Wilcoxon signed-rank test) post-injection. Recombination efficiency, as determined by the percentage of YFP^+^ brain microglia using flow cytometry, was 90.38 ± 2.98% (2 weeks) and 97.17 ± 1.49% (26 weeks) among viable doublet-excluded CD45^lo^CD11b^+^ cells. Data are presented as mean $\pm$ S.E.M. **e** Representative confocal images of F4/80^+^YFP^+^ hyalocytes (asterisks) and F4/80^−^YFP^+^ rMG in retinal whole mounts from *Cx3cr1*^*CreER*^*:Rosa26-YFP* at 2 weeks and 26 weeks after injection of TAM. Images are representative for four mice per timepoint. Scale bar = 50 µm

**Fig. 5:**
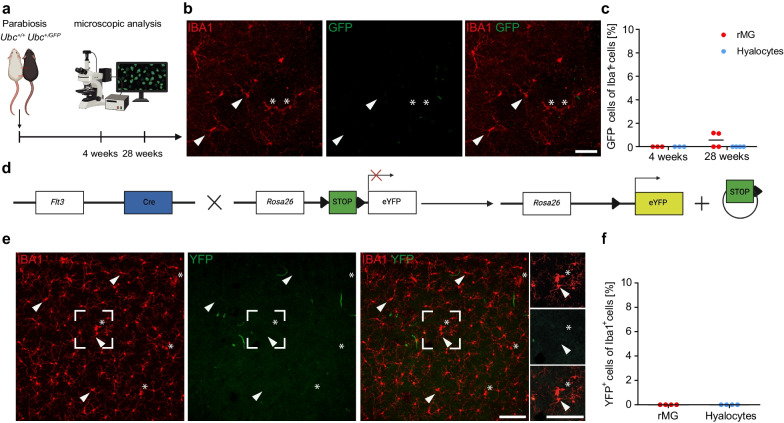
Circulating monocytes from adult hematopoiesis do not contribute to the resident hyalocyte pool under homeostasis.** a** Graphical scheme of parabiosis experiments. *Ubc*^*GFP/*+^ donor mice were surgically connected to *Ubc*^+*/*+^ wildtype mice for parabiosis and retinal whole mounts subsequently analyzed by fluorescence microscopy after 4 and 28 weeks, respectively. **b** Confocal images of retinal flat mounts from *Ubc*^+*/*+^ acceptor parabionts. Hyalocytes (asterisks) and rMG (arrowheads) in the inner plexiform layer can be identified. Images are representative for three mice (4 weeks) and four mice (28 weeks), respectively. Scale bar = 50 µm. **c** Quantification of GFP^+^ cells among IBA1^+^ hyalocytes and rMG in *Ubc*^+/+^ parabiotic mice 4 (N = 3) and 28 (N = 4) weeks after surgery. Each symbol represents one animal. Blood chimerism of CD11b^+^ myeloid blood cells in *Ubc*^+*/*+^ recipient parabionts, as assessed by flow cytometry, was 46.93 ± 5.6% for 4 weeks and 52.98 ± 6.9% for 28 weeks post surgery. **d** Graphical illustration describing the experimental setup. In *Flt3*^*Cre*^*:Rosa26-YFP* mice, constitutive activity of Cre-recombinase leads to YFP expression, under the control of the *Rosa26*-promoter, in all FLT3^+^ hematopoietic cells during fetal and postnatal hematopoiesis and their progeny. **e** Fluorescent microscopic visualization of IBA1 (red) and YFP (green) in hyalocytes (asterisks) and rMG (arrowheads) in *Flt3*^*Cre*^*:Rosa26-YFP* mice. Images are representative for four mice. Scale bar = 100 µm.** f** Quantification of YFP^+^ cells among IBA1^+^ hyalocytes and rMG in *Flt3*^*Cre*^*:Rosa26-YFP* mice (N = 4). Each symbol represents one animal from one litter

After having established a prenatal origin of murine hyalocytes, our next step was to analyze the contribution of circulating monocytes to the resident hyalocyte pool during postnatal development. Therefore, we took advantage of a postnatal fate mapping approach in which TAM is injected into six-week-old *Cx3cr1*^*CreER*^*:Rosa26-YFP* animals as described before [18, 56, 57] to label tissue-resident macrophages. Retinal flat mounts of these mice were analyzed 2 weeks and 26 weeks after injection (Fig. 4a, b). Hyalocytes (2 weeks: 95.93 ± 1.1, 26 weeks: 96.28 ± 1.1) showed, similar to rMG (2 weeks: 97.74 ± 0.5, 26 weeks: 98.01 ± 0.5), a high number of YFP^+^ cells (Fig. 4c, d), that remained stable over time resembling the low turnover rates observed in brain microglia, CAMs, and other ocular macrophages [15, 17–19]. Of note, the expression of F4/80 was suitable to discriminate between F4/80^+^ hyalocytes and rMG in *Cx3cr1*^*CreER*^*:Rosa26-YFP* mice (Fig. 4e). However, for the reason of comparability between both cell types and other ocular macrophages as well as previous studies [18], we decided to use Iba1 for the quantitative analysis going forward. To complement our data, we used parabiosis by surgically connecting *Ubc*^*GFP/*+^ mice, which express GFP under the control of the Ubiquitin C promoter in all cell types including monocytes in the blood stream, to *Ubc*^+*/*+^ mice and analyzed them 4 and 28 weeks after pairing (Fig. 5a). Indeed, GFP^+^IBA1^+^ hyalocytes were not found in the vitreous of the wildtype parabiont indicating a negligible contribution of the adult hematopoiesis to the hyalocyte pool under homeostatic conditions (Fig. 5b, c), whereas flow cytometry demonstrated a consistent level of blood chimerism in wildtype parabionts (4 weeks: 46.93 ± 5.6%, 28 weeks: 52.98 ± 6.9%). These results were further supported by the use of *Flt3*^*Cre*^*:Rosa26-YFP* mice, in which hematopoietic stem cells (HSCs) of the fetal and adult hematopoiesis and their progeny but not descendants of the YS hematopoiesis are labeled (Fig. 5d) [12, 22, 60]. In this model, YFP^+^ hyalocytes were not detected in the vitreous (Fig. 5e, f). All in all, these data support the notion that murine hyalocytes represent a long-living cell population, that seeds prenatally and is maintained through local self-renewal and without contribution of adult hematopoiesis.

### Hyalocyte maintenance depends on CSF1R signaling

**Fig. 6:**
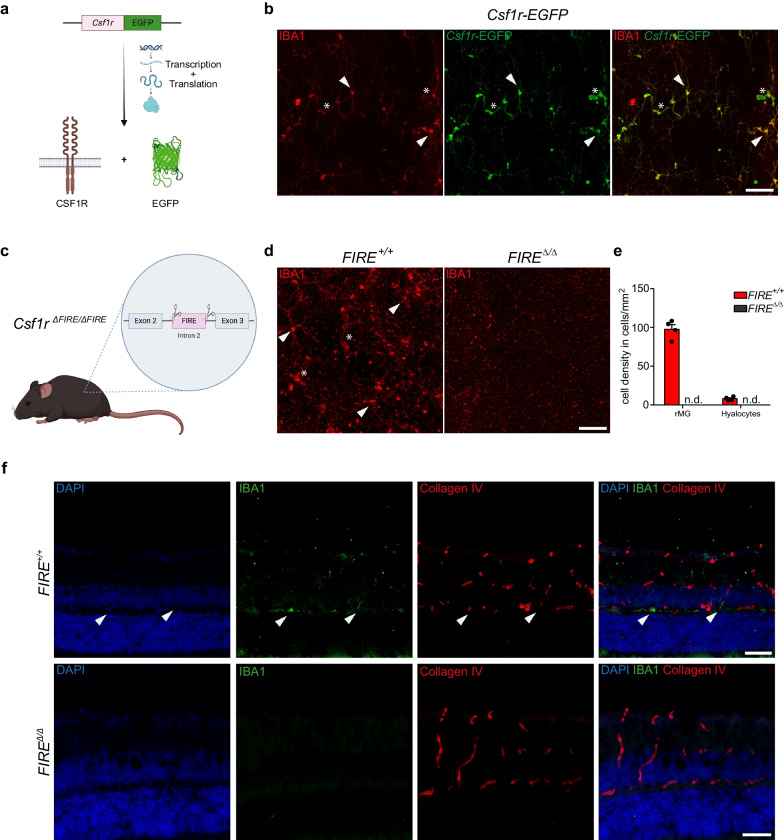
CSF1R-dependence of murine hyalocytes and retinal microglia.** a** Graphical scheme depicting the experimental setup. In *Csf1r-EGFP* mice, enhanced green fluorescent protein (EGFP) is expressed under the control of the transgenic *Csf1r* promoter. Subsequent protein biosynthesis leads to the simultaneous expression of CSF1R and EGFP in these mice. **b** Confocal images of IBA1 and anti-GFP immunofluorescence co-staining on retinal flat mounts from *Csf1r-EGFP* mice. EGFP^+^ hyalocytes (asterisks) and rMG (arrowheads) can be regularly identified. Images are representative for three mice. Scale bar = 50 µm. **c** Graphical illustration depicting the gene targeting approach in *Csf1r*^*∆FIRE/∆FIRE*^ mice. CRISPR/Cas9-based gene editing was applied to delete the *fms*-intronic regulatory element (FIRE) in the second intron of the *Csf1r* gene locus. **d** Confocal images of IBA1 immunofluorescence labeling on *Csf1r*^*∆FIRE/∆FIRE*^ mice and wildtype controls. Hyalocytes (asterisks) and rMG (arrowheads) can be found in wildtype mice, whereas IBA1^+^ myeloid cells are completely absent in *Csf1r*^*∆FIRE/∆FIRE*^ mice. Images are representative for four mice per group and two independent experiments. Scale bar = 50 µm**. e** Quantification of microglia and hyalocyte density in *Csf1r*^*∆FIRE/∆FIRE*^ (N = 4) and wildtype controls (N = 4). Data are presented as mean $\pm$ S.E.M**. f** Images from Collagen IV and IBA1 immunofluorescence co-staining on cryo-sections of eyes from wildtype controls (upper panel) and *Csf1r*^*∆FIRE/∆FIRE*^ mice (lower panel). Images are representative for four mice per group. Scale bar = 50 µm

Previous studies using pharmacological or genetic approaches to interfere with the function of CSF1R provided compelling evidence that most tissue-resident macrophages rely on sufficient stimulation of CSF1R, which is encoded by the *Csf1r* (historically known as *c-fms*) gene [61], through its ligands CSF1 or Interleukin-34 for their maintenance [62, 63]. To investigate whether murine hyalocytes also rely on CSF1R signaling for their perpetuation, we used *Csf1r-EGFP* transgenic MacGreen mice [64] to confirm CSF1R expression in these cells **(**Fig. 6a**)**. Indeed, we detected consistent transgene expression in murine hyalocytes and rMG, which, in the latter case, were previously found to express CSF1R [65–67] (Fig. 6b). After having confirmed CSF1R expression in hyalocytes, we sought to investigate whether an impairment of the CSF1R in macrophages would also result in alterations of hyalocytes in the murine vitreous. Therefore, we took advantage of a genetic approach using the recently generated *Csf1r*^*∆FIRE/∆FIRE*^ mouse line [68]. In these mice, CRISPR/Cas9-based gene editing was used to delete the *fms*-intronic regulatory element (FIRE), a superenhancer of the *Csf1r* gene locus [69] positioned in the second intron (Fig. 6c). Subsequent analysis revealed that several tissue-resident macrophages including microglia in the brain were depleted without causing the severe phenotype observed in *Csf1r-KO* mice [68]. Consequently, we hypothesized that, given the tight ontogenetic relationship between hyalocytes and microglia, ablation of FIRE would lead to reduced hyalocyte numbers in *Csf1r*^*∆FIRE/∆FIRE*^ mice. Immunofluorescence labeling and subsequent quantitative analysis in retinal whole mounts revealed a complete absence of hyalocytes and microglia in comparison to wildtype controls (Fig. 6d, e), thereby confirming our hypothesis.

Prenatally, the developing vitreous and lens rely on the hyaloid vasculature, which consists of the hyaloid artery, the vasa hyaloidea propria and the tunica vasculosa lentis, to meet their high metabolic demand [70]. Following the developmental growth phase, the metabolism of vitreous (and lens) decreases significantly which creates the need of a regression of the hyaloid vessels, mediated by murine hyalocytes as described in previous studies [71, 72], to avoid light scattering. To address the question, whether a depletion of murine hyalocytes is accompanied by a persistence of the hyaloid vasculature during adulthood, we analyzed cryo-sections of murine eyes from 4-month-old *Csf1r*^*∆FIRE/∆FIRE*^ mice and wildtype controls. Interestingly, FIRE ablation was, aside from the complete absence of myeloid cells in the retina and vitreous, not accompanied by morphological alterations of the vitreoretinal compartment. In particular, we did not find any sign of persistent hyaloid vessels or alterations of the superficial and deep plexus in the IPL and OPL (Fig. 6f). All in all, our data demonstrate that CSF1R is expressed by murine hyalocytes and rMG and that an intact FIRE sequence represents a prerequisite for the maintenance of these cell populations but is not associated with the persistence of the hyaloid vasculature during adulthood.

## Discussion

**Fig. 7:**
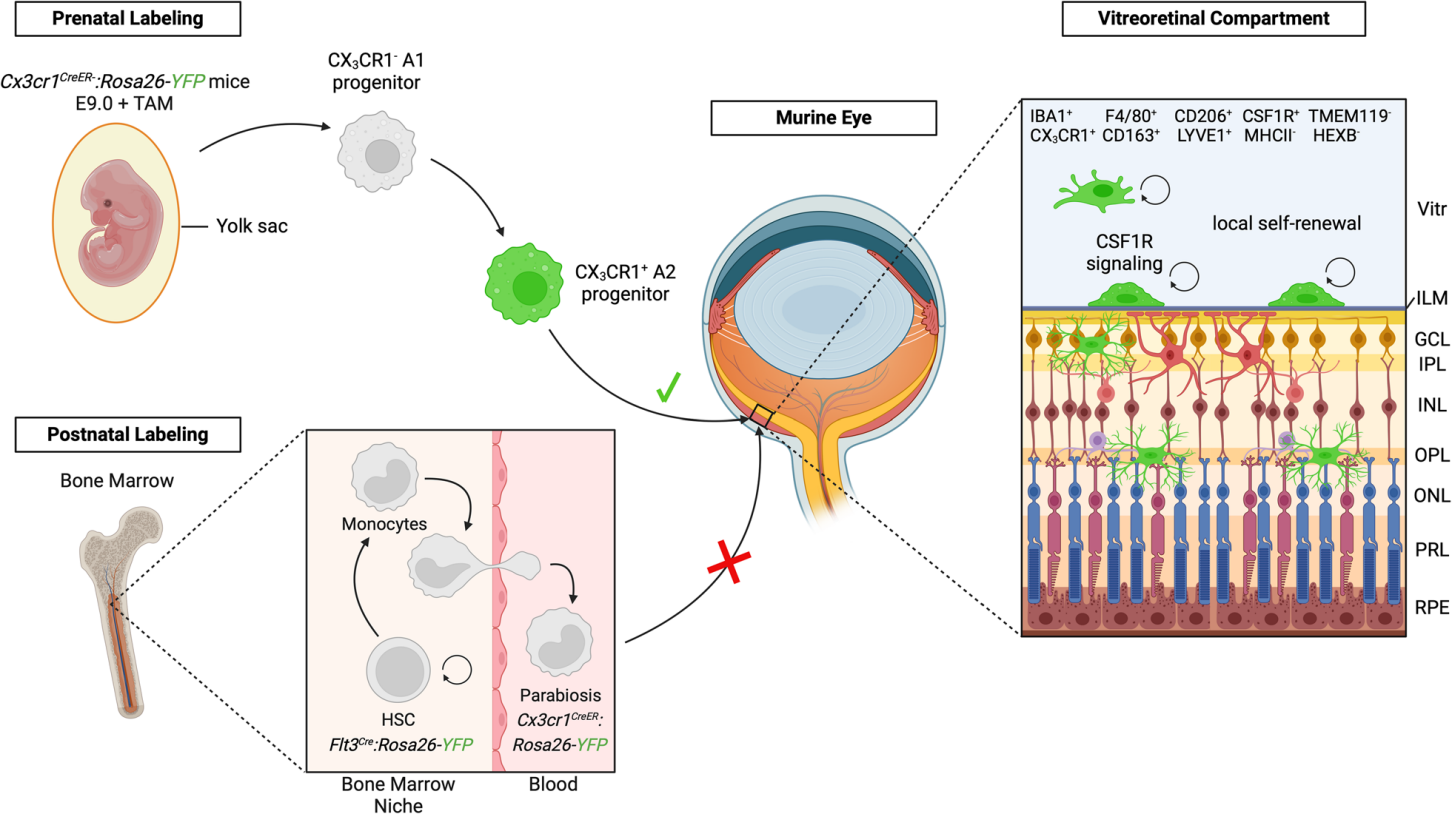
Origin, turnover and phenotype of murine hyalocytes. Graphical summary of the findings in this study. Prenatally, the local hyalocyte and microglial pool is recruited from yolk sac-derived CX_3_CR1^+^ A2 progenitors that are labeled with YFP (green) in *Cx3cr1*^*CreER*^*:Rosa26-YFP* mice after tamoxifen (TAM) injection at E9.0 and enter the vitreous cavity through the blood stream. Postnatally, fate mapping in adult *Cx3cr1*^*CreER*^*:Rosa26-YFP* mice has shown that hyalocytes, which exhibit a unique immunophenotype and rely on CSF1R-signaling for their maintenance, are long-living cells independent of replenishment from circulating peripheral myeloid cells from the definitive hematopoiesis. Hyalocytes are largely maintained by local self-renewal and reside above the inner limiting membrane while retinal microglia are located below in the neuroretina. The inner limiting membrane is constituted of both vitreal and retinal laminae. The vitreal side has a dense collagen fibril meshwork connected via extracellular matrices to the retinal glia limitans, built by the endfeet of the astrocytes (purple) residing in the nerve fiber and ganglion cell layer, separating hyalocytes and retinal microglia in two distinct compartments of the eye. *Vitr*—vitreous, *ILM*—inner limiting membrane, *GCL*—ganglion cell layer, *IPL*—inner plexiform layer, *INL*—inner nuclear layer, *OPL*—outer plexiform layer, *ONL*—outer nuclear layer, *PRL*—photoreceptor layer, *RPE*—retinal pigment epithelium

Here, we provide evidence that murine hyalocytes, the tissue-resident macrophages of the vitreous body, display a unique immunophenotype, originate from YS hematopoiesis and populate the developing eye as early as at E11.5. Within the vitreous, they establish a self-maintaining cell population, which persists until adulthood and relies on CSF1R for its maintenance, while being independent of blood monocytes (Fig. 7).

By performing immunofluorescence labeling on retinal flat mounts from *Cx3cr1-GFP* mice, we identified preretinal hyalocytes as CX_3_CR1^+^IBA1^+^F4/80^+^CD163^+^CD206^+^LYVE1^+^MHCII^−^HEXB^−^TMEM119^−^ cells confirming their established myeloid cell identity, as previously suggested by immunocytochemical studies in tissue-cultured hyalocytes [73] as well as in murine tissue sections [74]. Importantly, we show that murine hyalocytes exhibit a distinct immunophenotype in comparison to rMG, which consistently express both HEXB and TMEM119, as has already partially been described in previous studies [18, 47, 49, 50, 75, 76] including the work of Rajesh and colleagues employing the *Tmem119-GFP* mouse model to study tissue-resident macrophages at the vitreoretinal interface [77]. Over and above that, this corroborates the findings of recent studies investigating the transcriptional profile of human hyalocytes demonstrating that human hyalocytes express several myeloid signature genes [28], but concurrently display a unique gene expression profile in comparison to microglia in the human retina [78, 79]. The expression of CD163 and CD206 in hyalocytes might indicate similarities to CAMs in the brain, however, further research in necessary to support this hypothesis. Interestingly, homeostatic hyalocytes in the mouse do not express MHCII, which is in line with the findings of Vagaja and colleagues [74], whereas MHCII-expression was upregulated in murine models of peripheral lipopolysaccharide challenge and diabetic retinopathy suggesting a role of hyalocytes in the induction of adaptive immunity [74]. The eye, including the vitreous body, however, is regarded as an immune privileged site which means that proinflammatory stimuli cause attenuated immune responses [80, 81]. Given their strategical position at the vitreoretinal interface, hyalocytes might contribute to this phenomenon [48, 82, 83], in parts by the reduced expression of MHCII which is a known mechanism of immune privilege maintenance as shown for microglia in the CNS [84]. Interestingly, human adult hyalocytes regularly express *HLA-DR* under homeostatic conditions [28, 78, 85]. Therefore, the mechanisms by which the immune privilege of the eye is maintained and, specifically, the role that hyalocytes might play in different species still warrants further investigation. In addition, previous studies suggested ultrastructural differences between vitreoretinal hyalocytes and anterior hyalocytes residing in close proximity to the ciliary body [40]. Although the scope of this study was to investigate hyalocytes at the vitreoretinal interface, we acknowledge that single-cell profiling studies will be necessary to capture the complete, molecular heterogeneity of murine hyalocytes and to uncover differences between hyalocyte subpopulations in different anatomical localizations.

By performing immunofluorescence labeling on eyes at different stages of prenatal development, we found that myeloid cells were present in the periocular mesenchyme at E9.5 and hyalocyte precursors were identified in the developing vitreous cavity as early as E11.5. This is in line with studies examining the embryonic origin of microglia in the brain where precursors of microglia first colonized the adjacent mesenchyme at E9.0 and then entered the underlying neuroepithelium shortly after [11]. Our data indicate that myeloid cells may enter the developing eye through the optic stalk, as part of the developing CNS, which has been observed before in the context of rMG [86]. In this study, distinct waves of macrophages colonizing the murine retina were characterized, thereby confirming previous reports from studies in quails in which rMG tangentially populated the retina from central to peripheral areas and through radial migration from the vitreoretinal interface to the plexiform layers [87]. The presence of yet undefined myeloid cells at the vitreoretinal interface shortly after birth further suggests a common precursor of hyalocytes and rMG and favors a model in which immature rMG radially infiltrate the plexiform layers in the course of the ongoing stratification of the retina and undergo further maturation processes whereas hyalocytes remain and mature at the vitreal side of the interface [75, 86]. However, it is also imaginable that a bidirectional exchange between rMG and hyalocyte precursors occurs until the ILM is established, thereby separating these different microanatomical niches. Such a colonization pattern would strongly resemble the behavior of brain microglia and myeloid cells destined to become intracerebroventricular macrophages in the CNS [88, 89], but requires further investigation in the eye. Finally, the anatomical peculiarities of the eye need to be considered as the ciliary zonules connecting the ciliary body and the lens were recently identified as a possible trafficking pathway for immune cells [90], given the fact that a recent study suggested that the ciliary body may also serve as a source for rMG repopulation in a microglia cell depletion paradigm [91].

Previous studies investigating the origin and fate of murine hyalocytes have been restricted to bone marrow transplantation techniques relying on whole-body irradiation that may cause artificial conditions under which the engraftment of peripheral monocytes into tissues is facilitated [36–38, 92, 93]. Indeed, we found an engraftment of GFP^+^ cells in *Actb*^*GFP/*+^→*Actb*^+*/*+^ bone marrow chimeras with a morphology strongly resembling hyalocytes, which is in line with previous bone marrow transplantation studies reporting a significant engraftment of peripheral myeloid cells into the retina [18, 34, 38, 92]. To challenge the previous conception, we used an established inducible fate mapping model employing *Cx3cr1*^*CreER*^*:Rosa26-YFP* mice [15, 18, 56, 57], which labels CX_3_CR1^+^ A2 progenitors by TAM injection at E9.0 and their descendants [11, 13] to trace the engraftment of YS-derived macrophages into different ocular compartments in the absence of an artificial influx of peripheral immune cells. We observed recombination rates in murine hyalocytes that are comparable to those for other YS-derived macrophages [15, 18, 59]. These data point towards a prenatal origin of murine hyalocytes [20]. Furthermore, as the injection of TAM into the maternal body of *Cx3cr1*^*CreER*^*:Rosa26-YFP* mice at E9.0, given its limited bioavailability, does not lead to a labeling of HSCs in the FL [59], our approach is specific for and clearly demonstrates the major contribution of YS hematopoiesis to the hyalocyte population. Besides, the *Flt3*^*Cre*^*:Rosa26-YFP* mouse line used in this study was shown to label HSC descendants of the fetal and adult hematopoiesis [12, 15, 22, 60] and, given the absence of YFP^+^ hyalocytes in this model, further supports a YS-derived origin of murine hyalocytes and argues against a significant contribution of HSC-derived FL hematopoiesis.

Concerning the turnover of macrophages, different organs show remarkable differences. Whereas microglia in the CNS are long-living cells [15, 18] with random and limited self-expansion under homeostatic conditions [94], ocular macrophages in the choroid, cornea and ciliary body have at least in part been shown to be replenished by circulating monocytes [17, 18]. Using the *Cx3cr1*^*CreER*^*:Rosa26-YFP* mice in an adult fate mapping approach, we show that murine hyalocytes represent a long-living cell population, which remains stable over time. This is in line with a recent independent study by Rajesh and colleagues demonstrating that vitreous macrophages retain YFP-labeling over a period of 4 weeks after TAM-injection in adult *Cx3cr1*^*CreER*^*:Rosa26-YFP* mice [77]. However, in a previous study, we were able to demonstrate that it may last up to 4 weeks after injection until peripheral monocytes lose their YFP-label [57]. Furthermore, given the fact that distinct compartments of the eye and brain, such as the cornea and the choroid plexus, show a slow but steady turnover over months [15, 18], the assumption can be made that a time window of 4 weeks might be too short to use the full potential of this line [15, 18]. Therefore, our work provides a significant and necessary extension of the findings in the study by Rajesh and colleagues and allows to verify the hypothesis that hyalocytes are a self-maintaining cell population over an extended period of time. Moreover, our data illustrate that the maintenance of murine hyalocytes is independent of peripheral monocytes as shown by using parabiotic mice with a shared blood circulation. Finally, we further corroborated the independence of hyalocytes from adult hematopoiesis using *Flt3*^*Cre*^*:Rosa26-YFP* mice labeling FLT3-expressing HSCs [95], in which we did not observe a contribution of HSCs to the hyalocyte population. Taken together, these data clearly demonstrate that hyalocytes are a self-maintaining cell population under homeostatic conditions. Of note, vitreous detachment can often be observed at the human vitreoretinal interface which may impede local self-renewal in vitreoretinal hyalocytes and still requires further investigation. Furthermore, previous studies linked hyalocytes to several pathological conditions of the vitreoretinal interface like diabetic retinopathy [74, 96, 97] or proliferative vitreoretinopathy [27, 98–100]. These diseases may cause severe alterations in the local microenvironment which may ultimately lead to an additional influx of peripheral immune cells. In the retina, microglia represent the predominant immune cell population in several retinal disease models [17, 18, 101–106]. In the vitreous, however, the intravitreal injection of the proinflammatory chemokine CCL2 was found to increase the number of preretinal Ly6C^+^ monocytes [77] indicating that the myeloid compartment may undergo significant changes in its composition during disease. Therefore, future studies using tools to differentiate monocytes from resident hyalocytes, as the *Ccr2*^*CreER*^ mouse line [107], will be necessary to dissect the contribution of distinct cell populations to pathologies of the vitreoretinal interface. The translation of these findings into the human setting could open new therapeutic avenues as demonstrated for Osteopontin in neovascular AMD [108, 109], which is also expressed by hyalocytes on the transcriptional level [28, 78]. Moreover, aging is also associated with changes in long-living tissue-resident macrophages due to immunosenescence that may affect their responses to pathological insults, as already demonstrated for microglia in the brain [110, 111] and the retina [112]. In this context, targeting long-lived resident hyalocytes to reverse a potential immunosenescence-associated hyalocyte dysfunction for the treatment of vitreoretinal diseases could be achieved by local, intravitreal application of pharmacological agents, a practice performed in a daily manner in the field of ophthalmology.

Finally, we confirmed that hyalocytes, but also rMG, express CSF1R and, using *Csf1r*^*∆FIRE/∆FIRE*^ mice, we showed that CSF1R deletion in hyalocytes and rMG leads to complete absence of these cells in the murine eye. To our knowledge, this is the first report using this model to investigate macrophages in the eye and our results corroborate the findings of previous studies in which the deletion of the *FIRE* sequence in the *Csf1r* gene led to the depletion of several tissue-resident macrophage populations including brain microglia [68, 88, 113]. In the past, murine hyalocytes have been implied in the postnatal regression of the hyaloid vasculature [71, 72]. Although we found a complete absence of hyalocytes and rMG in *Csf1r*^*∆FIRE/∆FIRE*^ mice, we did not witness any morphological changes, such as persistent hyaloid vessels, in the eyes of these animals during adulthood. Of note, we examined eyes harvested from four-month-old animals. Therefore, we cannot exclude that hyalocytes play a relevant homeostatic role earlier and that the regression of the hyaloid vasculature is still delayed in *Csf1r*^*∆FIRE/∆FIRE*^ mice, which is compensated in their absence at later time points through hyalocyte-independent mechanisms [71, 72]. This warrants further investigation, as deeper knowledge of these mechanisms might be highly relevant for new therapeutic interventions for diseases such as persistent hyperplastic primary vitreous, an idiopathic eye disease of childhood characterized by an absent regression of hyaloid vessels [114, 115].

## Conclusions

In conclusion, our study reveals hyalocytes as a new member of the family of long-living, YS-derived tissue-resident macrophages, which are independent of a steady turnover by peripheral myeloid cells and rely on CSF1R for their maintenance in vivo. These data shed new light on the innate immune system of the eye and may pave the way for the development of new myeloid cell-focused therapies of vitreoretinal diseases adapted to the distinct anatomical niches of the eye.

### Supplementary Information

**Additional file 1: Figure S1. **Expression of CD163 in tissue-resident macrophages at the vitreoretinal interface. Representative images from immunofluorescence labeling for CD163 with IBA1 in *Cx3cr1-GFP* mice. Hyalocytes (asterisks) consistently show CD163 expression whereas rMG (arrowheads) are negative for this marker. Images are representative for three mice. Scale Bar = 50 µm.

**Additional file 2: Figure S2. **Immunophenotype of murine hyalocytes during prenatal development. Representative images from immunofluorescence labeling for CD11b, F4/80 and Isolectin B4 on *Cx3cr1-GFP* mice at E14.5. Noteworthily, Isolectin B4 also stains hyaloid vessels (arrow). Scale bar = 50 µm.

## Data Availability

The datasets used and/or analysed during the current study are available from the corresponding author on reasonable request.

## Abbreviations

AMD: Age-related macular degeneration
BSA: Bovine serum albumin
CAM: CNS-associated macrophage
CCL2: C–C chemokine receptor ligand 2
*Ccr2*: C–C chemokine receptor type 2
CSF1R: Colony Stimulating Factor 1 Receptor
CX_3_CR1: Fractalkine receptor
DAPI: 4′,6-Diamidine-2-phenylindole
E: Embryonic day
EMP: Erythromyeloid precursor
FBS: Fetal bovine serum
FIRE: *fms*-Intronic regulatory element
FL: Fetal liver
GCL: Ganglion cell layer
GFP: Green fluorescent protein
HSC: Hematopoietic stem cell
ILM: Inner limiting membrane
INL: Inner nuclear layer
i.p.: Intraperitoneal
IPL: Inner plexiform layer
ISP: Isotonic Percoll
L: Lens
LP: Lens placode
LYVE1: Lymphatic vessel endothelial hyaluronic acid receptor 1
mes: Mesenchyme
MHCII: Major histocompatibility complex II
Nb: Neuroblast
n.ep.: Neuroepithelium
ONL: Outer nuclear layer
OPL: Outer plexiform layer
OS: Optic stalk
OV: Optic vesicle
P: Postnatal day
PBS: Phosphate buffered saline
PFA: Paraformaldehyde
PRL: Photoreceptor layer
rMG: Retinal microglia
RT: Room temperature
RPE: Retinal pigment epithelium
TAM: Tamoxifen
Vitr: Vitreous body
WT: Wildtype
YFP: Yellow fluorescent protein
YS: Yolk sac
